# Supplementary figures and images for: Quantitative modeling of signaling in aggressive B cell lymphoma unveils conserved core network
Source: PLoS Comput Biol. 2024 Oct 1;20(10):e1012488. doi: 10.1371/journal.pcbi.1012488 (PMC11469524; doi:10.1371/journal.pcbi.1012488)

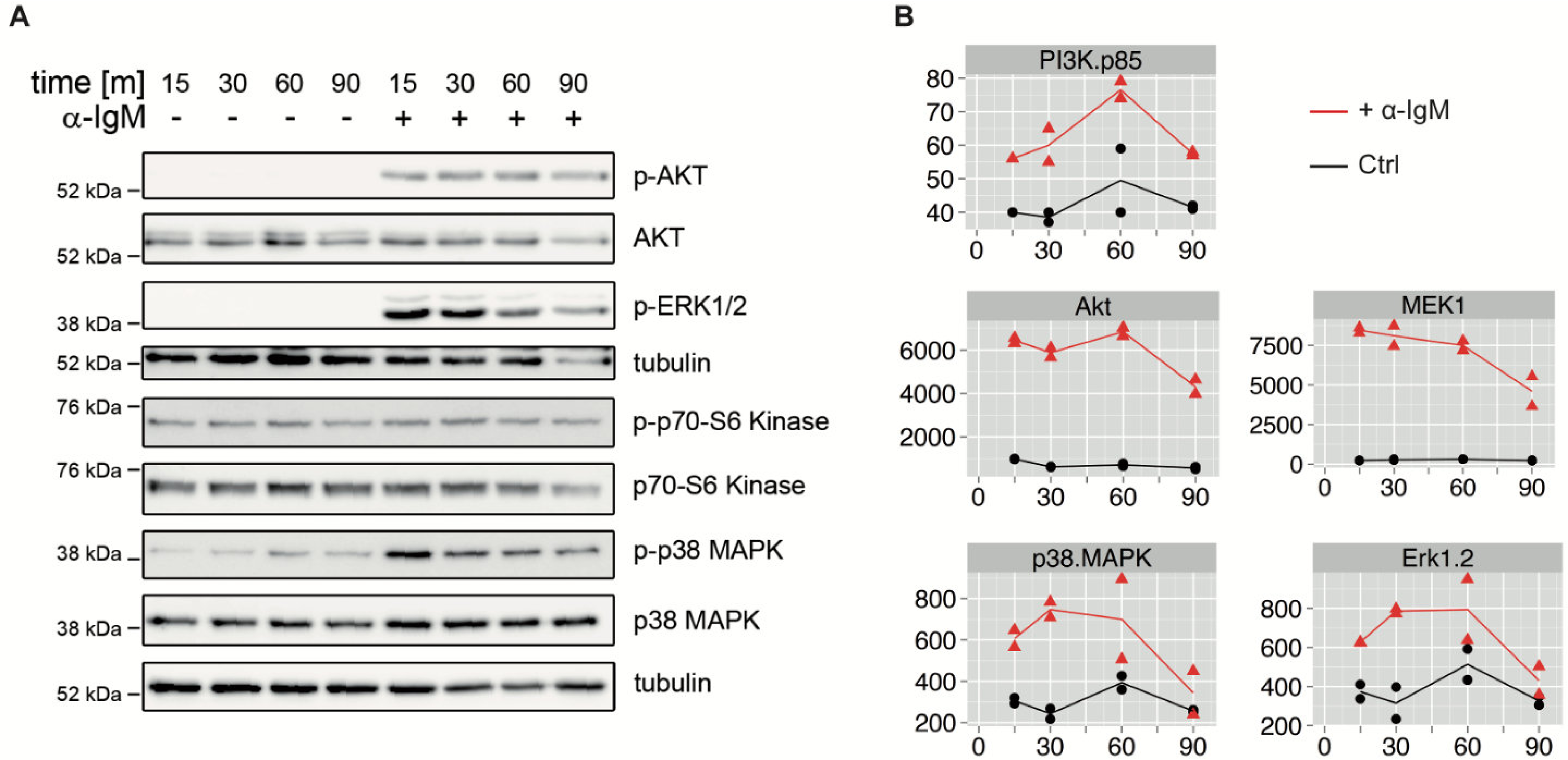

Supplement: S1 Fig — The phosphorylation changes were estimated by (A) immunoblot and (B) Bead-based ELISA analysis on the same samples depicted in fluorescence intensities. (TIF) [file pcbi.1012488.s001.tif]

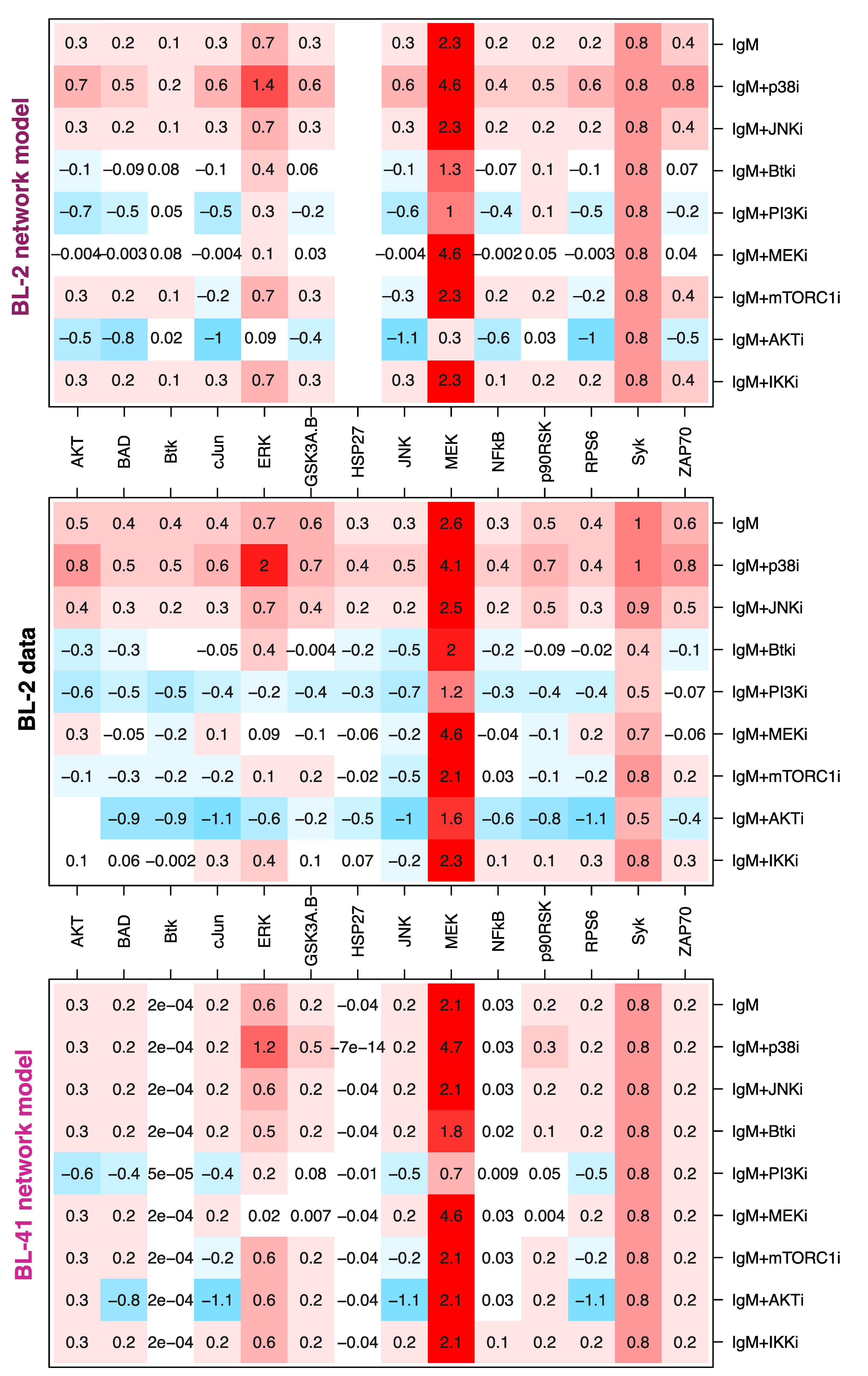

Supplement: S2 Fig — Heatmaps of mean log2 fold changes (n = 3) to untreated for model input data for BL-2 (BL-2 data) and model results for best network structures found for BL-2 and BL-41 data when adjusting the response coefficients but not the network structure to BL-2 data. Blanks in BL-2 data are withhold data which act contrary to central model assumptions that inhibitors reduce phosphorylations of downstream targets not phosphorylation of their targets and are therefore not modelled (see Material and Methods). Blanks in BL-2 model are due to the removal of the edge connecting to HSP27 in the final model structure. For more information see S1 Text BL-2_network_model.html: Tab ‘11. Rem. PI3K -> Btk’ and BL-41_network_model.html: Tab ‘Model transfer to BL-2’. (TIF) [file pcbi.1012488.s002.tif]

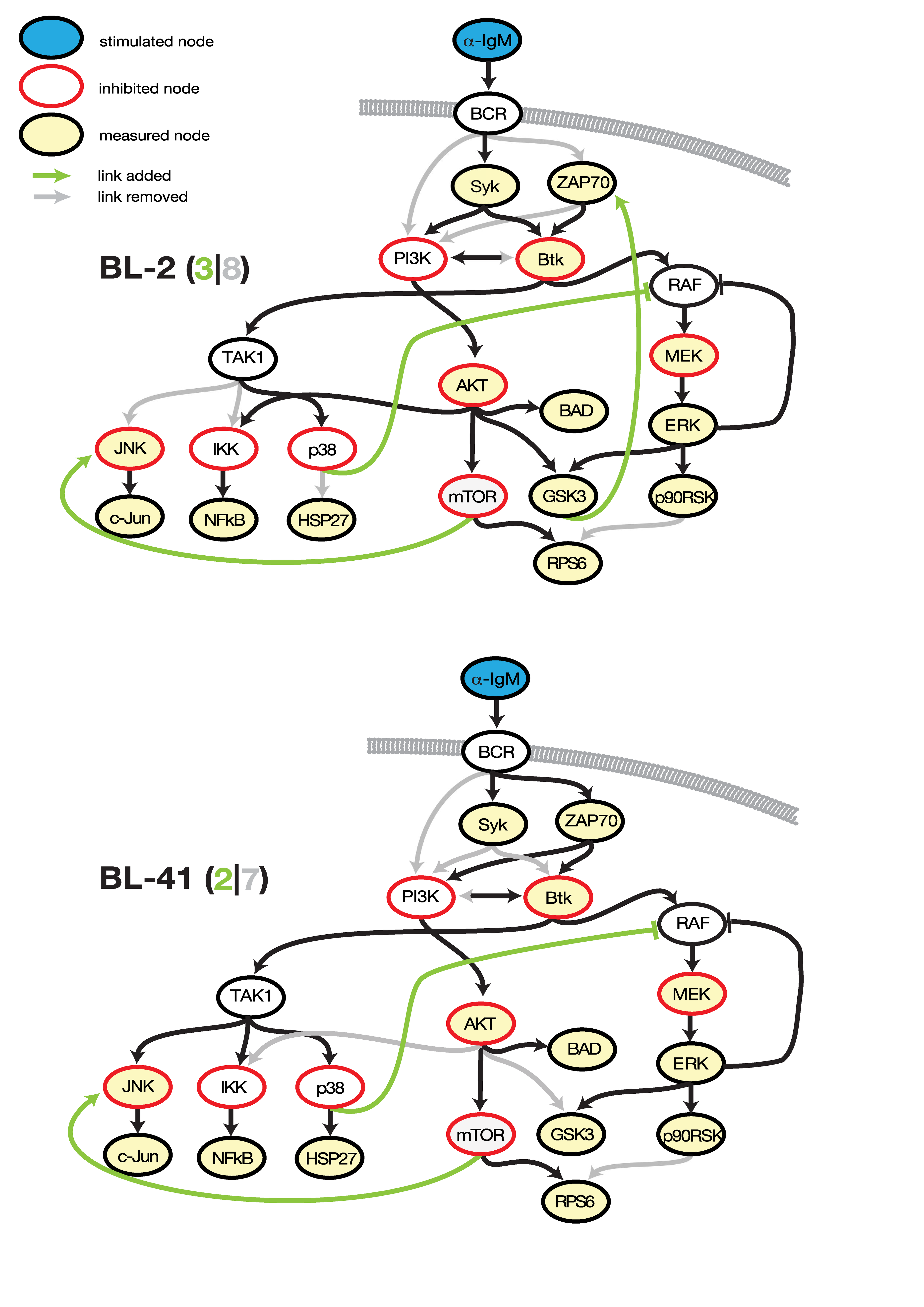

Supplement: S3 Fig — Literature-derived starting network (cf. Fig 1C) and adjustments during model development for individual models for the indicated cell lines. Numbers indicate added (green) and removed (grey) links. (TIF) [file pcbi.1012488.s003.tif]

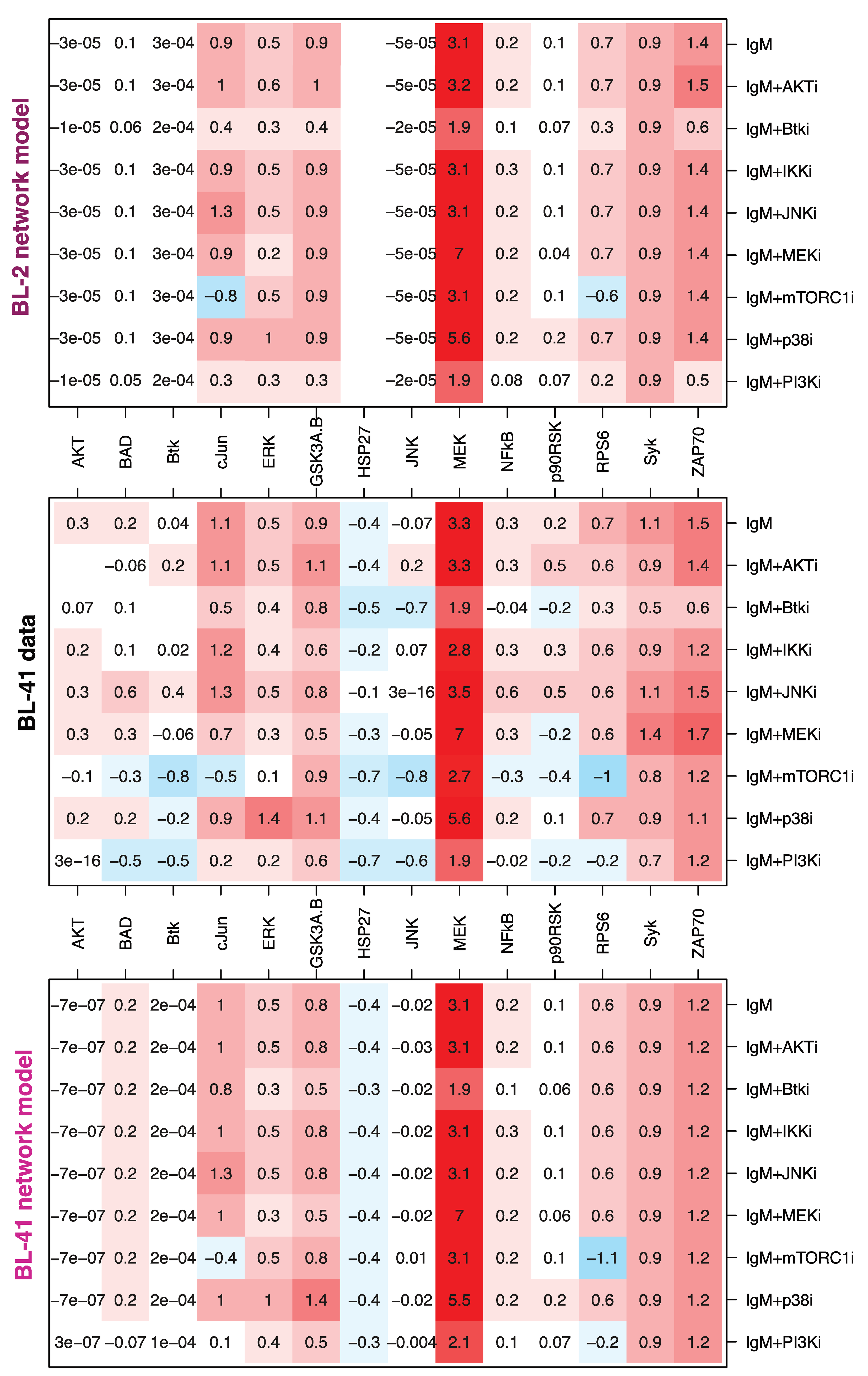

Supplement: S4 Fig — Heatmaps of mean log2 fold changes (n = 3) to untreated for model input data for BL-41 (BL-41 data) and model results for best network structures found for BL-2 and BL-41 data when adjusting the coefficient (path)s but not the network structure to BL-41 data. Blanks in BL-41 data are withhold data which act contrary to central model assumptions that inhibitors reduce phosphorylations of downstream targets not phosphorylation of their targets and are therefore not modelled (see Materials and Methods). Blanks in BL-2 model are due to the removal of the edge connecting to HSP27 in the final model structure. For more information see S1 Text BL-41_network_model.html: Tab ‘9. Rem. Syk -> PI3K’ and BL-2_network_model.html: Tab ‘Model transfer to BL-41’ variant BL2-model as initial model. (TIF) [file pcbi.1012488.s004.tif]

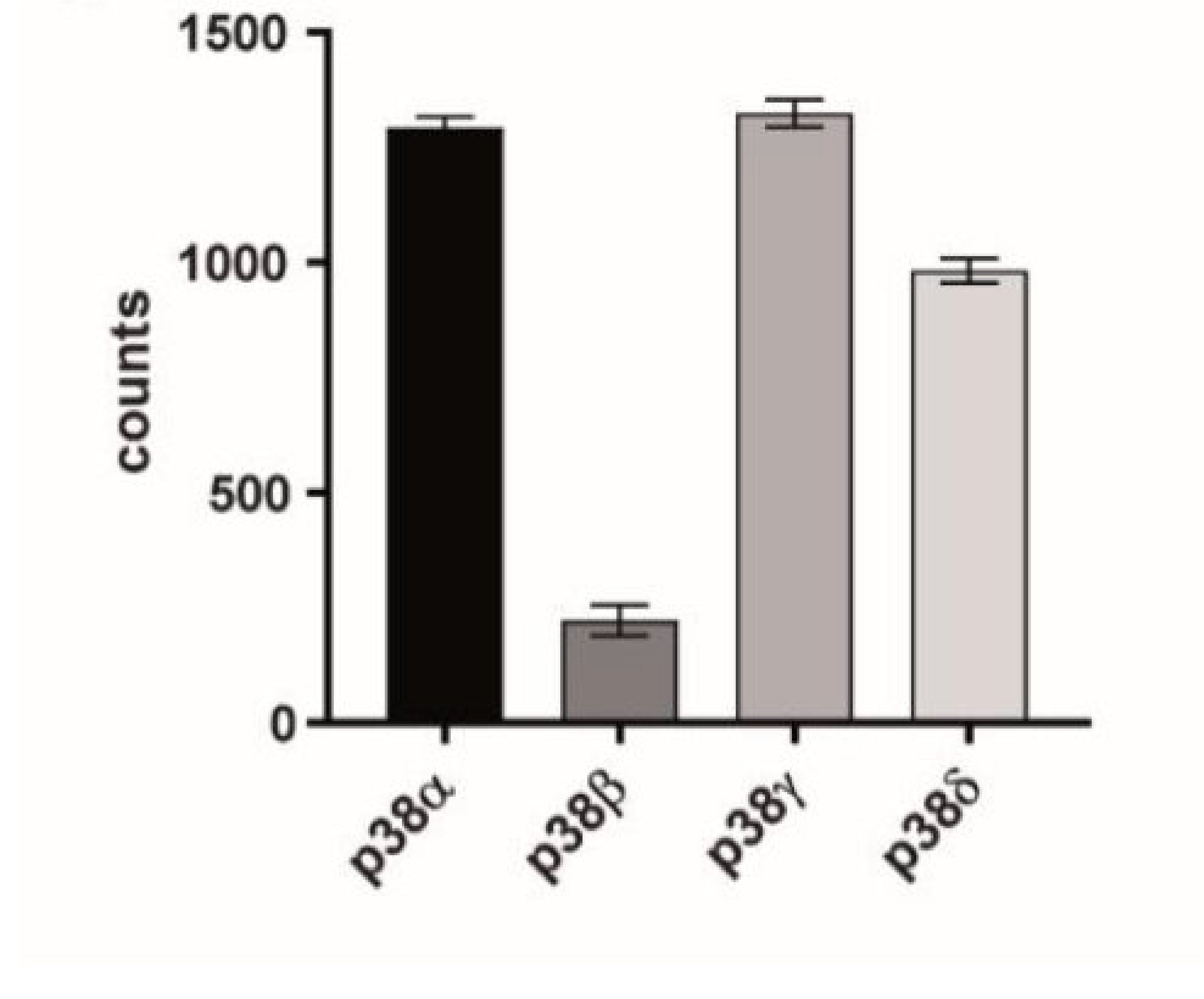

Supplement: S5 Fig — Total reads of the p38 subunits α (MAPK14), β (MAPK11), γ (MAPK12), δ (MAPK13) are displayed from RNA sequencing analysis of BL-2 cells (n = 3, data from microarrays conducted at [64]). (TIF) [file pcbi.1012488.s005.tif]

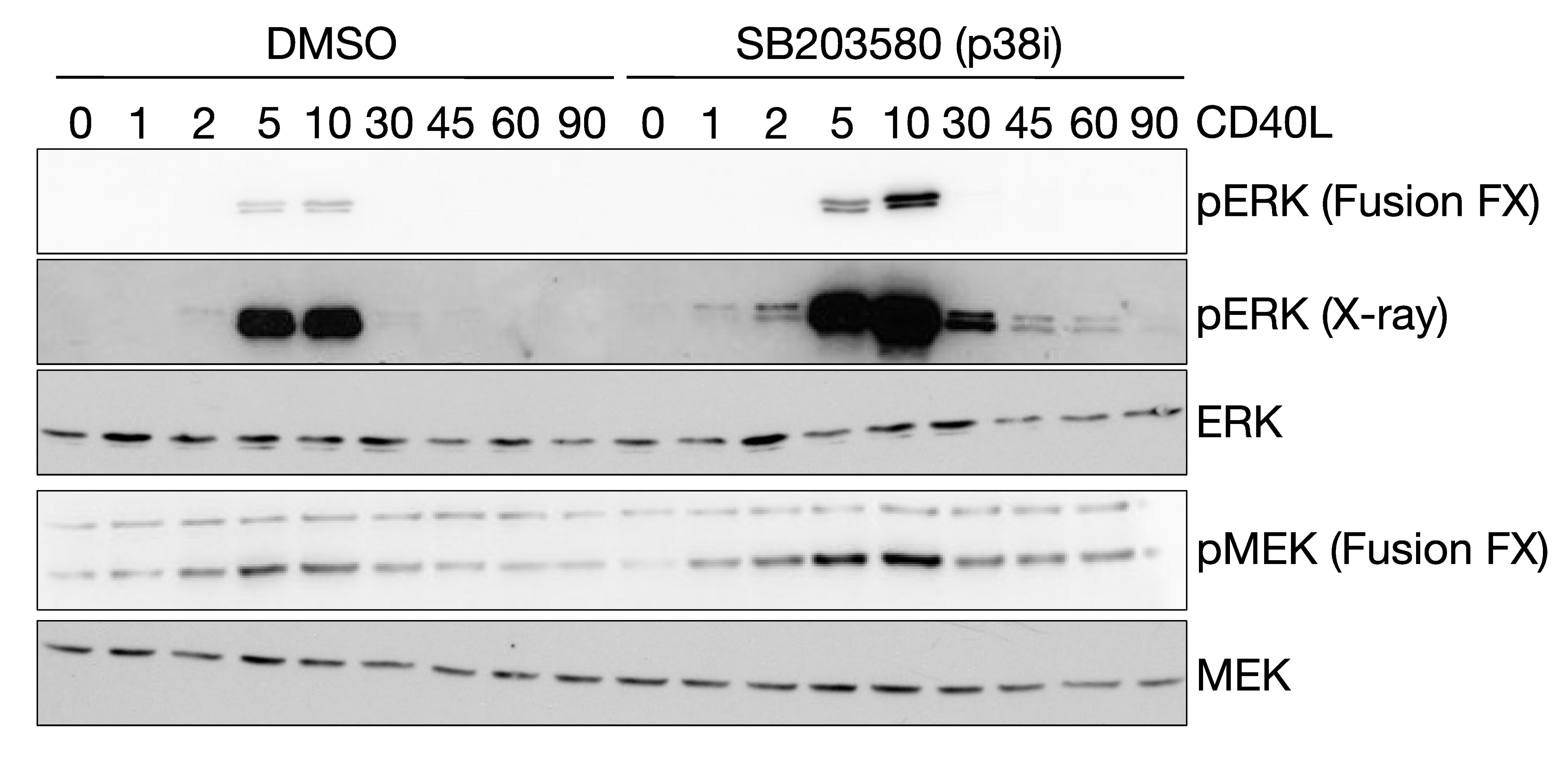

Supplement: S6 Fig — CA-46 BL cells were treated with CD40 ligand for up to 90 min without or with 2μM p38 inhibitor SB203580. Data were analyzed by classical IB-chemoluminescence imaging and by the Fusion-FX platform. (TIF) [file pcbi.1012488.s006.tif]

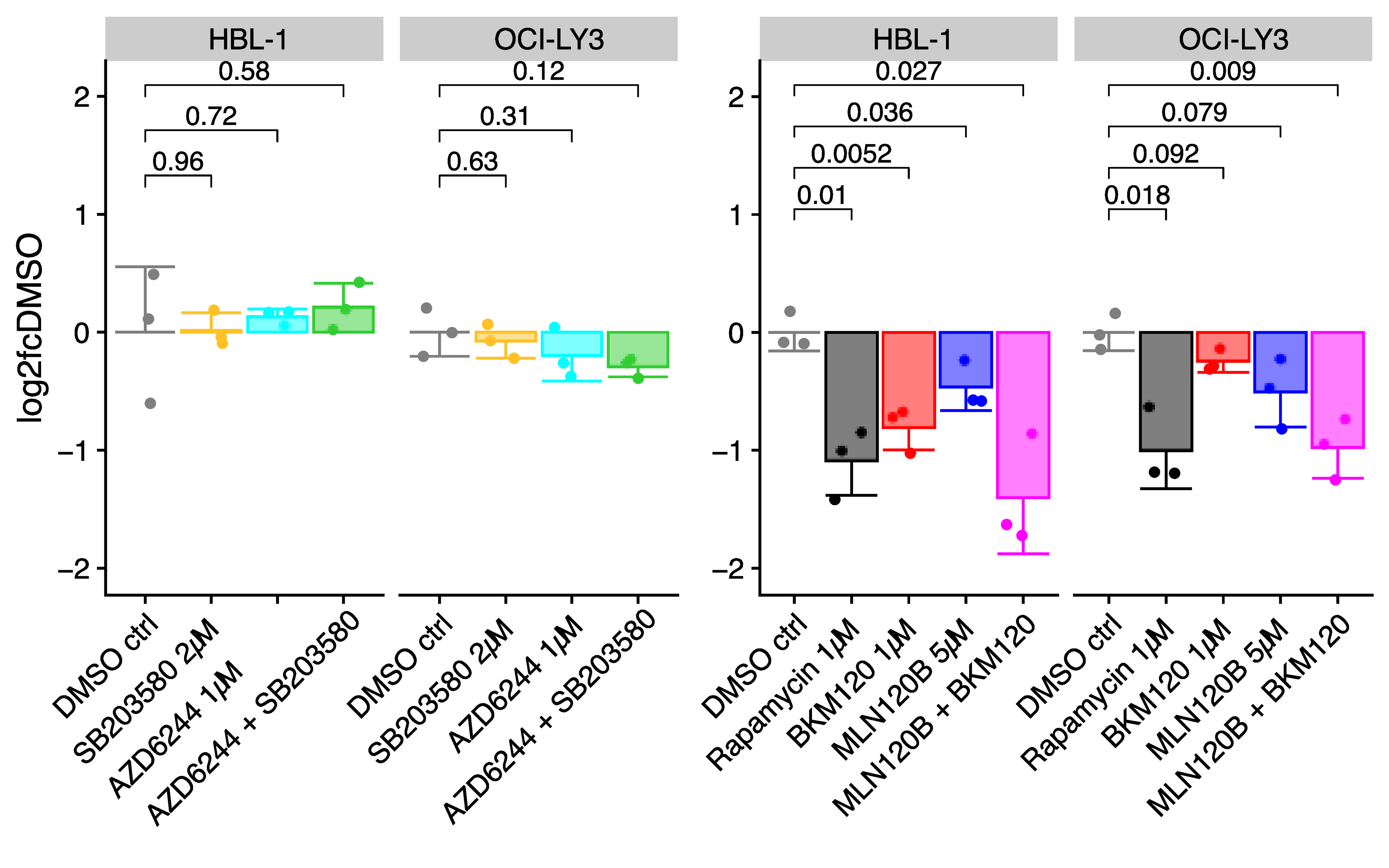

Supplement: S7 Fig — Cell counts of indicated DLBCL cell lines treated for 72h with indicated inhibitors and concentrations, as log2 fold change to respective mean solvent control (DMSO). P-values derived by two-sided T-test; n = 3. Inhibitors (target): SB203580 (p38), AZD6244 (MEK), Rapamycin (mTORC1), BKM120 (PI3K) and MLN120B (IKK). (TIF) [file pcbi.1012488.s007.tif]
