# Supplementary material for: Quantitative modeling of signaling in aggressive B cell lymphoma unveils conserved core network
Source: PLoS Comput Biol. 2024 Oct 1;20(10):e1012488. doi: 10.1371/journal.pcbi.1012488 (PMC11469524; doi:10.1371/journal.pcbi.1012488)
Supplement: S1 Table — Parametrization for the best fit and in brackets the upper and lower boundaries of the 95% confidence interval, derived by profile likelihood (alpha = 0.05, 1 degree of freedom). ni denotes nonidentifiable confidence intervals in that direction (i.e., alteration of the coefficient can be compensated by changing other model coefficients). Coefficient (path)s with non-overlapping confidence intervals are termed significantly different between BL-2 and BL-41. Coefficient term definition: r_source_target. (DOCX) [file pcbi.1012488.s008.docx]

| **Coefficient (path)s** | **BL-2** | **BL-41** | **Significant difference** |
| --- | --- | --- | --- |
| r_BAD_AKT | 0.69 (0.4 –1.24) | -0.08 (-0.22 – 0.06) | YES |
| r_GSK3A.B_AKT | 0.37 (0.18 –0.74) | -0.5 (-0.58 – -0.43) | YES |
| r_IKK_AKT | 2.52 (0.09 – ni) | 0.12 (0.04 – 0.19) | NO |
| r_mTORC1_AKT | 2.63 (0.12 – ni) | -0.49 (-0.59 – -0.42) | YES |
| r_BCR_ α-IgM*r_Syk_BCR | 0.52 (0.43 – 0.6) | 0.64 (0.54 – 0.72) | NO |
| r_PI3K_Btk | 5.36 (0.67 – ni) | 19.83 (17.39 – 23.1) | NO |
| r_MEK_Raf*r_Raf_Btk | 107.52 (76 – 233) | 169.28 (159 – 180) | NO |
| r_MEK_Raf*r_p38_Btk*r_Raf_p38 | -32.86 (-43 – -22) | -75.93 (-85 – -65) | YES |
| r_GSK3A.B_ERK | 0.27 (0.11 – 0.44) | 0.0019 (-0.11 – 0.1) | YES |
| r_MEK_Raf*r_Raf_ERK | -9.13 (-15.7 – -4.8) | -9.17 (-14.5 – -7.6) | NO |
| r_p90RSK_ERK | 0.36 (0.16 – 0.57) | 0.22 (-0.24 – 0.67) | NO |
| r_ZAP70_GSK3A.B | 1.2 (0.71 – 2.13) | 1.54 (1.26 – 1.81) | NO |
| r_NFkB_IKK | 0.22 (0.08 – ni) | -1.01 (-1.62 – -0.42) | YES |
| r_cJun_JNK | 0.91 (0.57 – 2.43) | 25.81 (3.59 – ni) | YES |
| r_ERK_MEK | 0.3 (0.23 – 0.36) | 0.18 (0.15 – 0.21) | YES |
| r_AKT_PI3K | 1.58 (0.45 – ni) | -1.17 (-1.41 – -1.02) | YES |
| r_Btk_Syk | 0.1 (0.02 – 0.14) | 0.05 (0.03 – 0.06) | NO |
| r_PI3K_Syk | -0.56 (-2.29 – -0.05) | -0.21 (-0.42 – -0.01) | NO |
| r_Btk_ZAP70 | 0.12 (0.07 – 0.19) | 0.03 (0.01 – 0.03) | YES |
| r_JNK_mTORC1 | 0.35 (0.1 – ni) | 0.04 (0.03 – 0.04) | YES |
| r_RPS6_mTORC1 | 0.31 (0.08 – ni) | 0.8 (0.63 – 0.97) | NO |
