# Supplementary material for: Quantitative modeling of signaling in aggressive B cell lymphoma unveils conserved core network
Source: PLoS Comput Biol. 2024 Oct 1;20(10):e1012488. doi: 10.1371/journal.pcbi.1012488 (PMC11469524; doi:10.1371/journal.pcbi.1012488)
Supplement: S2 Table — Parametrization for the best fit and in brackets the upper and lower boundaries of the 95% confidence interval, derived by profile likelihood (alpha = 0.05, 1 degree of freedom). ni denotes nonidentifiable confidence intervals in that direction (i.e., alteration of the coefficient can be compensated by changing other model coefficients). Coefficient (path)s with non-overlapping confidence intervals are termed significantly different between HBL-1 and OCI-LY3. – indicates missing link in either cell line. Coefficient term definition: r_source_target. (DOCX) [file pcbi.1012488.s009.docx]

| **Coefficient (path)s** | **HBL-1** | **OCI-LY3** | **Significant difference** |
| --- | --- | --- | --- |
| r_BAD_AKT | 2.23 (1.78 – 2.67) | 2.5 (1.61 – 3.42) | NO |
| r_GSK3A.B_AKT | 1.51 (1.31 – 1.67) | -0.27 (-0.27 – -0.2) | YES |
| r_IKK_AKT | 3.02 (2.35 – 3.68) | 9.8 (9.39 – 10.04) | YES |
| r_mTORC1_AKT | 2.62 (2.38 – 2.91) | 7.51 (7.13 – 7.88) | YES |
| r_p90RSK_AKT | 2.34 (1.89 – 2.78) | **–** | **–** |
| r_PI3K_Btk | 0.84 (0.72 – 0.96) | 1.3 (1.3 – 1.36) | YES |
| r_MEK_Raf*r_Raf_Btk | 1.83 (1.27 – 2.39) | 2.39 (2.39 – 5.3) | YES |
| r_MEK_Raf*r_Raf_ERK | -2.92 (-4.02 – -2.14) | -9.24 (ni – -5.71) | YES |
| r_Btk_GSK3A.B | 1.59 (1.36 – 1.8) | 2.8 (2.5 – 2.94) | YES |
| r_NFkB_IKK | 0.67 (0.52 – 0.81) | 0.22 (0.13 – 0.31) | YES |
| r_cJun_JNK | 1.89 (1.66 – 2.11) | 2.71 (2.48 – 2.83) | YES |
| r_ERK_MEK | 0.44 (0.31 – 0.62) | 0.6 (0.39 – 0.6) | NO |
| r_AKT_PI3K | 0.14 (0.11 – 0.17) | 0.0036 (0.0036 – 0.02) | YES |
| r_MEK_Raf*r_Raf_p38 | -2.22 (-2.74 – -1.71) | 0.16 (-0.43 – 0.86) | YES |
| r_AKT_cJun | 0.15 (0.12 – 0.18) | **–** | **–** |
| r_JNK_mTORC1 | 0.64 (0.57 – 0.7) | 0.25 (0.25 – 0.26) | YES |
| r_RPS6_mTORC1 | 1.9 (1.75 – 2.04) | 1.25 (1 – 1.57) | YES |
| r_p90RSK_ERK | **–** | 1.65 (0.99 – 2.24) | – |
| r_AKT_IKK | **–** | 0.1 (0.1 – 0.11) | – |
| r_GSK3A.B_cJun | **–** | 0.28 (0.28 – 0.29) | – |
