# Supplementary material for: Quantitative modeling of signaling in aggressive B cell lymphoma unveils conserved core network
Source: PLoS Comput Biol. 2024 Oct 1;20(10):e1012488. doi: 10.1371/journal.pcbi.1012488 (PMC11469524; doi:10.1371/journal.pcbi.1012488)
Supplement: S1 Text — (https://zenodo.org/doi/10.5281/zenodo.10732059): BL-2_network_model.html. BL-41_network_model.html. DLBCLs_network_model.html. global_extend.R. (DOCX) [file pcbi.1012488.s010.docx]

**Overview of Online Supplements (**https://zenodo.org/doi/10.5281/zenodo.10732059**):**

**>BL-2_network_model.html:**

**Network derivation BL-2:** Step-by-step modeling workflow using R package STASNet (https://github.com/molsysbio/STASNet) to model BL-2 perturbation data from the literature-guided starting model (initial model) until the final model (11. Rem. PI3K -> Btk). To illustrate the modeling workflow, analysis and decisions for each modeling step we provide all necessary input files and function commands used during the modeling workflow as they would have been used inside R for the initial model. We further illustrate for all modeling steps four aspects:
 1. *Visualization* – description of the model

- network graph
 - parameter heatmap – quantification of the coefficient path(s), i.e., model parameters

2. *Goodness of fit*

- ranked likelihood of top performing initializations + antibody-wise coefficient of determination (R^2^)
 - side-by side heatmaps of data and best fit simulation
 - quantile-quantile plot of residuals versus a normal distribution
 - side-by side heatmaps of unseen data and best fit simulation

3. *Network alterations* – information for further model development

- nonessential links, list of links that would insignificantly contribute to the model fit ($\alpha=0.05$)
 - link extensions, list of single links that significantly would improve the model fit ($\alpha=0.05$).
 - profile likelihood – parameter sensitivity analysis

4. *Conclusion* – reasoning about next modeling step as single link removal or addition depending on 3.

**Model transfer to BL-41:** Transfer of best BL-2 model to model BL-41 data in three variants:

1. *BL2-model as initial model* – allow all coefficients to adapt to BL-41 data
2. *BL2-model as initial model fixed inhibitor (to BL2*) – fix inhibitor quantification to BL-2 values and allow all other coefficients to adapt to BL-41 data
3. *BL2-model as initial model fixed inhibitor (to mean of BL2 and BL41) –* fix inhibitor quantification to mean of BL-2 and BL-41 values and allow all other coefficients to adapt to BL-41 data

**>BL-41_network_model.html:**

**Network derivation BL-41:** Step-by-step modeling workflow using STASNet to model BL-41 perturbation data from the literature-guided starting model (initial model) until the final model (9. Rem. Syk -> PI3K). All code and step illustrations as described for BL-2_network_model.html above.

**Model transfer to BL-2:** Transfer of best BL-41 model to model BL-2 data:

1. *BL41-model as initial model* – allow all coefficients to adapt to the BL-2 data

**>DLBCLs_network_model.html:**

**Initial models:** Comparative modeling results for HBL-1 and OCI-LY3 cells when starting from the literature-derive network structure (literature) or from the best-found structure for modeling BL-2 data (BL2).

**HBL1- development:** Step-by-step modeling workflow using STASNet to model HBL-1 perturbation data. All code and step illustrations as described for BL-2_network_model.html above.

**OCI-LY3 development:** Step-by-step modeling workflow using STASNet to model OCI-LY3 perturbation data. All code and step illustrations as described for BL-2_network_model.html above.

**Fitting summary:** Collection of model fitting statistics (number of parameters, residuals, WSSR for each modeling step) and visualization.

**Model transfer:** Transfer of best HBL-1 model structure to model OCI-LY3 data and *vice versa*.

**>global_extend.R:**

Convenience script to rerun **STASNet** R package fitting function *createModel()* for every possible extendable link with a provided number of starting parametrizations. The default method for calculating the initializations is set to ‘geneticlhs’ from the **lhs** R package. Run ./global_extend.R --help for usage description.
