# Supplementary material for: Quantitative modeling of signaling in aggressive B cell lymphoma unveils conserved core network
Source: PLoS Comput Biol. 2024 Oct 1;20(10):e1012488. doi: 10.1371/journal.pcbi.1012488 (PMC11469524; doi:10.1371/journal.pcbi.1012488)
Supplement: Attachment — Submitted filename: Replies_to_reviewers.pdf [file pcbi.1012488.s011.pdf]

Comments to the Authors:

Reviewer #1: The authors present new models of B cell lymphoma using their Boolean Nested Effects Modelling (B-NEM) approach. Compared to previous models of BCR signaling, the presented models have more ample scope, i.e. covers multiple pathways and their cross talks, and is (semi-)quantitative, therefore allowing to better study also complex network mechanisms such as feedback and cross-talks. The model is derived starting from literature-derived networks and is trained using in house targeted phosphoproteomics (magnetic bead-based multiplex immunoassay) data to add and remove links and estimate parameter values using their R package STASNet. The most impactful result of the paper is that, using computational modeling on two Burkitt lymphoma cell lines, the authors identify important regulatory mechanisms not yet described in BCR activated signaling. These pathways crosstalks were extensively validated and characterized experimentally using westerns blot and global phosphoproteomics. Additionally, the authors identify a core network that is shared also with Diffuse large B cell lymphoma cell lines, while also highlighting some differences. Overall, these results can help us to better understand B cell lymphoma aberrations with possible future clinical implications. This is a very interesting paper with a very rigorous modeling development and impactful biological insights.

1. I might not be reading the data right, but why does the MEK inhibitor in the presence of IgM stimulation induce MEK activation in all 4 cell lines (e.g. for BL-2 in stimulated condition MEK has activity = 2.6 in the uninhibited condition and 4.6 in the MEKi condition)? For BL-41 this is the case also for the unstimulated condition (MEK activity in MEKi = 0.3)

Answer: We and others previously observed a negative feedback from ERK to (C)RAF (Dougherty et al., 2005; Fritsche-Guenther et al., 2011). As a consequence, inhibition of MEK causes an inactivation of ERK and in turn a reduction of the feedback inhibition on RAF. This unhinged RAF activity in turn leads to increased MEK phosphorylation by a constantly active RAF. In our study, the corresponding MEK activation is, as the reviewer rightly pointed out, observed also in lymphoma cells. In our model the causative feedback for this accumulation of active MEK is encoded by the coefficient path r_MEK_Raf*r_Raf_ERK (cf. **Supplemental Table S1** and **S2**). This coefficient path is strongly negative in all four modelled cell lines. This is illustrated in **Figure 2E**, **Figure 5D** and the novel **Figure 6** as well as **Supplemental Tables S1** and **S2** in more detail. Furthermore, we added an explanation of the MEK hyperactivation in the discussion on **page 33**.

2. Would be interesting to investigate what are the main differences between the BL-2 and BL-45 data (e.g. with a simple scatterplot, and possibly also with the DLBLC cell lines). The BL-41 data seems easier to predict: the reduced chi-square for the BL-2 even after BL-2 specific training (adjusted) is worse than the one obtained for BL-41 with the original literature network. Do the author know what makes the BL-2 data more difficult to predict? It would be interesting to visualize also the model fit to each experimental condition (as supplementary).

Answer: This is an excellent suggestion to directly compare the different cell lines and their models. We address this in the following way:
(i) To get a better comparison between all four cell lines, we now include a qualitative comparison of the four models for the core network. This is now summarized in the new **FIGURE 6**.
(ii) We added new **Suppl. Figures 2** and **4** for BL-2 and BL-41cells that allows to directly compare data and models as heatmaps, including the transfer models from the respective other cell line. This is linked to **FIGURE 1** and **2** and discussed in the main text (**pages 16, 17, 19**)
(iii) We added to the discussion on **page 17** our interpretation of why BL-2 structure is better transferable than BL-41: "We assume that the reason for the better transferability is that the BL-2 perturbation data show a higher complexity (above noise) than the BL-41 data, in general and especially for the very central AKT readout. This allows the more complex BL-2 learned network structure to also fit the less complex BL-41 data, but not *vice versa* (compare **Suppl. Figures S2** and **S4**)."

3. I understand why the authors use the unstimulated data as 'test' set when looking separately at performances of the BL-2 and BL-41 models to ensure consistency. However, if the aim is to have the best model that can generally describe BL, those data could provide very useful for model training. Since the authors are anyway testing generalizability of the trained models on different cell lines, I would find it interesting to train the models also with the full data (stimulated and unstimulated) for each cell line.

Answer: We did not include both data sets for two reasons:
(i) In preliminary modeling attempts of BL-2 we have fitted both data sets by one model and realized that the model fit was qualitatively not better but worse. The reason for this is that we doubled the number of data points with low dimensional data (all signals down or not regulated) and thereby reducing the weight on specific stimulus-only observations. Two such examples are the upregulation of only MEK upon MEK inhibition as only

readout to explain the ERK-RAF feedback as well as the p38 crosstalk to RAF/MEK/ERK causing upregulation of ERK and MEK which is only strongly visible in the presence of an upstream stimulus. Therefore, we decided it best to use the data with low complexity only as a sanity check to prevent over-fitting. We mention the complexity difference now in the manuscript on **page 14**.
(ii) Due to the DLBCL cell lines exhibiting constitutively active BCR signaling we termed the models better comparable when restricting model fitting to the same data set , i.e., the BCR-stimulated data set only.

4. Do I understand correctly that no replicates were performed for the Bio-Plex multiplex immunoassay? Can the authors be confident that the differences between cell lines is not driven by measurement error?

Answer: We completely agree that replicate measurements are essential as a reliable data source. Therefore, for all Bio-Plex perturbation experiments three biological replicates have been conducted, of which we show the mean values in the figures. We use the standard-deviation to estimate the error models for the fitting procedure. We now explicitly indicate the number of replicates more clearly in the caption for the respective **Figures** (**1A, 2A, 2F** and **5A**).

5. Why is the added negative feedback from p38 to RAF1 instead of directly to MEK? I would imagine that for the model if it difficult to distinguish between the two since RAF1 is not perturbed nor measured in the data used for model training. Wouldn't the data in Figure 3 suggest that the feedback mechanism is not mediated by RAF1?

Answer: The current model cannot resolve where exactly the crosstalk between p38 and RAF/MEK acts and thus the term for the p38 crosstalk is given as the combination of response coefficients: "r_MEK_Raf*r_p38_Btk*r_Raf_p38" in BL-cells and „r_MEK_Raf*r_Raf_p38" in DLBCLs (cf. **Supplementary Table S1** and **S2**). The model is underdetermined at this point due to the experimental design defined by the antibody sets available for the Bio-Plex/Luminex assay platform and used inhibitors. This underscores the reviewer's presumption that the models quantification would not be different when modelling from p38 directly to MEK as each individual coefficient (i.e., r_MEK_Raf or r_Raf_p38) is structurally nonidentifiable, due to RAF neither being measured nor perturbed. The automatic identifiability analysis in STASNet identifies those nonidentifiable coefficients and combines them to identifiable combinations.
We extend the description of the combination of coefficients in the manuscript at **page 5** and in the methods section at **page 8-10**. Furthermore, the identifiable coefficient path(s) as the fitted parameters are now explicitly mentioned in **Figure 1B**.

We further improved the discussion by arguing why we do not rule out RAF to be the target of the crosstalk and that model-wise MEK is an equally likely target on **page 32:**
„RAF is a complex kinase family with more activity regulating phosphosites than we could observe with our analysis and at least another important isoform BRAF (Lavoie & Therrien, 2015). Therefore, we cannot rule out that the effect could still manifest on other RAF1 activity sites or the BRAF isoform. As RAF activity is neither measured nor perturbed, the model coefficients of the p38 crosstalk are only identifiable as combinations with the RAF->MEK link (**Suppl. Table 1** and **2**) which means a direct crosstalk from p38 to MEK would result in the same fit."

Minor:

6. Since MRA is not broadly known, it would be useful to have a short section describing the principles of the MRA, e.g. what is the intuition behind the model formalisms, what are the model parameters.

Answer: The model parameters are coefficients/ identifiable coefficient path(s) made of local network connections(local response coefficients), and perturbation coefficients (stimulus and inhibitor coefficients).
We now describe this aspect and our version of MRA briefly in the introduction on **page 5** and the materials and methods section on **pages 8-10**.

7. How are inhibitors effects simulated? In page 8 the authors specify that "fixed the parameters encoding the inhibitor strengths" but never mention how simulations of these models intuitively work and/or what are the model parameters.

Answer: Local response coefficients and inhibitors are related in a multiplicative manner. For example, the effect of an AKT inhibition on downstream target BAD is modelled as the product of the AKT inhibitor response coefficient times the local response coefficient from AKT to BAD. To simulate a perturbation effect from AKT inhibition on BAD (in the absence of feedbacks and other connections from AKT to BAD) one would multiply the inhibitor coefficient with the local response coefficient, never an inhibitor coefficient alone. Fixing the inhibitor coefficient to a certain value causes the model to compensate for this in the remaining coefficients. Biologically speaking all cells have been treated with the same inhibitor concentration. If they respond differently not the

inhibitor coefficient should be different but the network coefficients. By fixing the former we enforce this and enable a better comparability between cell line models. We introduced this technique in Dorel et al. 2021 Figure 3A (https://doi.org/10.1371/journal.pcbi.1009515).
We now reference this study when we fix the inhibitor coefficients together with a brief explanation at **page 19**. The material and methods section also now contains a description of inhibition modeling on **page 9** and a previously present paragraph for fixing of inhibitor coefficients on **page 10**.

8. Check order of citation of Figures, e.g., figure 1A never cited in text, panel D cited before panel C

Answer: **FIGURE 1A** is now cited in the main text on **page 14**. **FIGURE 1D** is now referenced after **FIGURE 1C**.

9. On page 20 the authors cite FIGURE 3F instead of FIGURE 3E

Answer: We have corrected this in the revised manuscript accordingly.

Reviewer #2: The manuscript describes modeling of BCR signaling using systematically perturbed phosphorylation data in two types of cells: BL-2 and BL-41 cells. The authors use the software package STASNet (Steady-State Analysis of Signalling Networks) that was described by the team of Nils Blüthgen in 2018. This package is based on Modular Response Analysis (MRA) method that was developed by Kholodenko's team to infer the response network from the steady-state global responses to perturbations. The authors start from literature-based models and use perturbation data for one cell line to create a model for two cell lines and reveal network features of the BCR signaling network, validated experimentally using phospho-proteomics assays. The manuscript is well-written and provides a good biological background and motivation for modeling and analysis.

Two major issues are:
- Not enough background on the modeling techniques used, and some explanations are not clear.
- Supplemental web pages provide a lot of additional information clarifying the modeling and analysis process, but these pages are not described in detail and not referred to from the manuscript in a standard way (e.g. Figure S10, etc).

Answer: To further improve the manuscript, we added the following information:
(i) more details on the modelling techniques (Introduction **page 5** and methods **pages 8-10**) as explained also to reviewer 1 minor comment 6 and 7.
(ii) a supplemental section '**Overview of Online Supplements**' that describes the supplementary web pages is added in order to describe the organization of the online content giving an impression how the step by step modelling strategy was conducted. We now also refer to the supplemental web pages within the manuscript at corresponding paragraphs (**Figure 1, 2, 5** and accompanying text).

Specifically:
1. The word semi-quantitative modeling is never defined. Even the notion of steady state modeling is never mentioned. I had to go all the way down to Kholodenko et al., 2002 to understand what is semi-quantitative. The authors need at least one paragraph describing the terms semi-quantitative modeling (it's not commonly used), MRA, and STASNet. Figure 1A-B is a nice introduction but it is not enough, a figure like Fig .2 in Dorel et al., 2018 would be helpful to understand the underlying mechanism and what are parameters in the model.

Answer: We now added a concise description of MRA terms in the introduction on **page 5** as well as in the methods section on **page 8** hopefully in a way not to plagiarize our or other's previous work on MRA/STASNet.

2. The authors never describe what are parameters to be fitted, and they use the terms "model parameters", "strength parameters", "network coefficients" and "pathway coefficients" without any explanation. Is it the same as "response coefficient" in STASNet? It needs to be defined and used consistently.

Answer: We apologize that the wording seemed to be not as specific as necessary. All references to model parameters we now term "coefficients" and the identifiable combination of coefficients we term "coefficient paths". We introduce the term's meanings in the main text at **page 5** and in the methods section at **pages 8-10**.

3. The authors use the word "stronger". Is it equivalent to "larger in absolute value"? Specifically, they say "the parameter corresponding to the pathway from Btk via p38 to MEK is estimated to be stronger in BL-41 than in BL-2 cells". Is stronger in this sentence means larger in absolute value (it may be negative)?

Answer: Indeed, in here the word "stronger" is used as larger in absolute value. In the referenced sentence we refer to a negative response coefficient, indicating inhibitory action, which can be stronger or weaker depending

on its value. We have now added the interpretation of (local response) coefficients in the main text at **page 5** in the introduction and in the description for novel **Figure 6.**

4. “Unseen nodes” need to be better described. It’s really confusing: the authors have some nodes being unmeasured, and some nodes being measured but not used for network construction.

Answer: To the best of our knowledge, we only refer to “unseen data” never to an unseen node, which we agree would be confusing. Maybe the reviewer could point out where we used this term?
Regarding the measured values that have not been used for modelling those are the measurements of AKT phosphorylation upon AKT inhibition and Btk phosphorylation upon Btk inhibition, because the apparent inhibitor mode of action is not as mandated by our modeling method, i.e., to act downstream of the kinase and not at the kinase itself. Since this explanation was missing, we have included this explanation to the legend of the new **suppl. Figure 2** and **4** that show data and best fit for the model learned final network structures. We also included this explanation in the methods section on **page 10**.

5. Model/network adaptation with sequential adjustment steps are apparently described in the supplemental web pages. It would be much clearer if the authors directly refer to the webpage “BL-2 network model development” when talking about 7 and 5 adjustments, etc. For example, the paragraph on page 10 states: “During model adaption, 4 adjustments were shared between BL-2- and BL-41. This includes, two of the three additional links already identified in BL-2 cells before, i.e., p38->RAF and mTORC1->JNK. However, 7 and 5 adjustments were BL-2- and BL-41- specific, respectively (FIGURE 2C). This divergence in proposed network structure also manifests in the fact that only 14 of the 21 identifiable path coefficients were shared in both cell lines.” It’s absolutely not clear what are these 7 adjustments and what are these 5 adjustments. The figure 2C is just a Venn diagram with numbers 0,1,2,5 and 6. What are these 14 path coefficients is not clear. The webpage describes 31 significant link additions when the network is transferred to BL-41 cell line, but these 31 links do not correspond to anything in the manuscript.

Answer: We are grateful for this suggestion. We now refer more directly to the supplemental web pages/tabs. We also added a new supplementary **Figure 3** which shows the network changes made for individually modelling of BL-2 and BL-41 data. Thus, the number of adjustments and the reasoning of shared and unshared links is directly evident.
A side note on the html interpretation of the significant extension parts: this lists all link additions that would significantly improve the current model, as the next single added link, not all at once. This aspect is now also explained in the **Overview of Online Supplements** section in the appendix.

In conclusion, it’s a very interesting methodology, model, and results, but a better description of the modeling and analysis process is required. Perhaps supplemental material guiding through supplemental web pages could do the job.

Answer: We thank the reviewer again for this constructive point and we now include an overview description of the supplemental web pages in the supplement titled: ‘**Overview of Online Supplements**’.
